# Stability and Reproducibility Underscore Utility of RT-QuIC for Diagnosis of Creutzfeldt-Jakob Disease

**DOI:** 10.1007/s12035-015-9133-2

**Published:** 2015-04-01

**Authors:** Maria Cramm, Matthias Schmitz, André Karch, Eva Mitrova, Franziska Kuhn, Bjoern Schroeder, Alex Raeber, Daniela Varges, Yong-Sun Kim, Katsuya Satoh, Steven Collins, Inga Zerr

**Affiliations:** 1 Department of Neurology, University Medical Center Goettingen and German Center for Neurodegenerative Diseases (DZNE)—site Goettingen, Robert-Koch Str. 40, 37075 Göttingen, Germany; 2 Department of Prion Diseases, Slovak Medical University Bratislava, Limbová 14, 833-03 Bratislava, Slovakia; 3 Thermo Fisher Scientific, Prionics AG, 8952 Schlieren, Switzerland; 4 Ilsong Institute of Life Science, College of Medicine, Hallym University, Anyang, Republic of Korea; 5 Department of Molecular Microbiology and Immunology, Nagasaki University Graduate School of Biomedical Sciences, Sakamoto 1-12-4, Nagasaki, Japan; 6 Department of Pathology, The University of Melbourne, Parkville, 3010 Australia

**Keywords:** Cerebrospinal fluid, Creutzfeldt-Jakob disease, Diagnostic test, Prion protein, Real-time quaking-induced conversion

## Abstract

Real-time quaking-induced conversion (RT-QuIC) allows the amplification of miniscule amounts of scrapie prion protein (PrP^Sc^). Recent studies applied the RT-QuIC methodology to cerebrospinal fluid (CSF) for diagnosing human prion diseases. However, to date, there has not been a formal multi-centre assessment of the reproducibility, validity and stability of RT-QuIC in this context, an indispensable step for establishment as a diagnostic test in clinical practice. In the present study, we analysed CSF from 110 prion disease patients and 400 control patients using the RT-QuIC method under various conditions. In addition, “blinded” ring trials between different participating sites were performed to estimate reproducibility. Using the previously established cut-off of 10,000 relative fluorescence units (rfu), we obtained a sensitivity of 85 % and a specificity of 99 %. The multi-centre inter-laboratory reproducibility of RT-QuIC revealed a Fleiss’ kappa value of 0.83 (95 % CI: 0.40–1.00) indicating an almost perfect agreement. Moreover, we investigated the impact of short-term CSF storage at different temperatures, long-term storage, repeated freezing and thawing cycles and the contamination of CSF with blood on the RT-QuIC seeding response. Our data indicated that the PrP^Sc^ seed in CSF is stable to any type of storage condition but sensitive to contaminations with blood (>1250 erythrocytes/μL), which results in a false negative RT-QuIC response. Fresh blood-contaminated samples (3 days) can be rescued by removal of erythrocytes. The present study underlines the reproducibility and high stability of RT-QuIC across various CSF storage conditions with a remarkable sensitivity and specificity, suggesting RT-QuIC as an innovative and robust diagnostic method.

**Electronic supplementary material:**

The online version of this article (doi:10.1007/s12035-015-9133-2) contains supplementary material, which is available to authorized users.

## Introduction

Transmissible spongiform encephalopathies or prion diseases are characterized by the aggregation and accumulation of misfolded scrapie prion protein (PrP^Sc^) in brain tissue. They can occur spontaneously (sporadic) but also be due to familial and iatrogenic causes. Sporadic Creutzfeldt-Jakob disease (sCJD) is the most common prion disease in human, followed by genetic CJD (gCJD) and iatrogenic CJD (iCJD). Different molecular sCJD subtypes, which show unique clinicopathological phenotypes and transmission characteristics [1] and depend on the codon 129 genotype of the prion protein gene (*PRNP*) as well as the type of PrP^Sc^, have been reported [2, 3].

The adaptation of in vitro amplification systems for the detection of PrP^Sc^ in human cerebrospinal fluid (CSF) was an innovation for the pre-mortem diagnosis. RT-QuIC analysis uses recombinant prion protein (recPrP) as a substrate to amplify very small amounts of PrP^Sc^ seed in human CSF to detectable levels. Time course of conversion can be monitored in real-time by a fluorescent dye using a fluorescent reader. Four previous studies demonstrated the diagnostic potential of RT-QuIC through amplification of PrP^Sc^ from human CSF, derived from sCJD and gCJD patients [4–7].

However, despite these initial reports, establishing RT-QuIC as a routine diagnostic test in clinical practice requires a comprehensive validation and standardization. To date, there is a lack of studies dealing with inter-laboratory reproducibility and standardization, assessment of short- and long-term stability and contamination of CSF samples with potential inhibitory blood cells of the RT-QuIC seeding response in human CSF of CJD patients. All of these parameters may lead to an artificial decrease or increase of PrP seeding activity in CSF and to false positive or negative results.

The aim of the present study was to further analyse the applicability of RT-QuIC for routine diagnostic purposes. Analysis of 110 CSF samples from prion disease patients and 400 from a control panel consisting of 200 retrospectively tested patients (diagnoses including Alzheimer’s disease [AD], Parkinson’s disease [PD], inflammatory [IF] and other non-prion diseases) and 200 prospectively randomly selected patients was undertaken to obtain diagnostic data more aligned to a routine clinical setting. Also, two ring trials were performed to generate data about the reproducibility of the RT-QuIC assay. Finally, the influence of different CSF storage conditions (long- and short-term storage or repeated freezing and thawing cycles) and the influence of blood contamination on the RT-QuIC seeding response were examined.

## Materials and Methods

### Patients

CSF samples were collected through routine activities of the German National Prion Disease Surveillance Center associated with the University Medical Center Göttingen. Samples were derived from 110 prion disease patients, consisting of 64 sCJD, 39 gCJD (33 E200K, 6 V210I mutation carriers) and 7 FFI (D178N mutation), as well as 400 control patients. Initial data on the cohort have been published elsewhere [7]. The data were used to calculate the sensitivity and specificity. All prion disease cases were neuropathologically confirmed.

The 200 retrospectively tested control patients represent those with either a clinically or pathologically defined alternative diagnosis (92 female, 108 male; aged 16–87 years; mean age 64.1 ± 0.9 years at notification). This control group consisted of AD patients (rapid progressive and classical forms), patients with depression, Dementia with Lewy bodies, PD, psychosis, bipolar disorder, multiple sclerosis, epilepsy, schizophrenia, inflammatory CNS disease and others. The 200 prospective samples were enrolled from the Neurochemistry Laboratory at the Department of Neurology in the framework of quality assurance and were tested blinded for clinical and personal data. Gender- and age-dependent effects on the RT-QuIC seeding response were excluded before [7].

### CSF Samples

CSF samples were stored at −80 °C prior to analysis. Haemorrhagic CSF samples were excluded from the study.

### Ethical Statement

The study was conformed to the Code of Ethics of the World Medical Association and informed consent was given by all study participants or their legal next of kin with the study being approved by the local ethics committee in Goettingen (No. 24/8/12). All samples were analysed blinded for at least personal data.

### RT-QuIC Analysis

The RT-QuIC was performed as described previously [7]. Briefly, 85 μL of reaction buffer were seeded with 15 μL of freshly thawed and neat CSF to a final volume of 100 μL, with each CSF sample generally run in triplicate. Prepared plates were sealed (VWR, Hannover, Germany) and incubated in a plate reader (FLUOStar OPTIMA, BMG Labtech, Ortenberg, Germany) at 42 °C for 80 h with intermittent shaking cycles, consisting of 1 min double orbital shaking at 600 rpm followed by a 1-min break. Beta-sheet formation kinetic was determined by measuring the thioflavin T (ThT) fluorescent signal (450 nm excitation and 480 nm emission) every 30 min in relative fluorescence units (rfu). When we obtained more than 50 % positive replicates (signal ≥10,000 rfu after 80 h), the sample was considered as positive. Controls were considered as negative when more than 50 % of the replicates revealed a RT-QuIC response after 80 h below 10,000 rfu.

### Expression and Purification of Recombinant Sheep-Hamster PrP

All RT-QuIC experiments were performed using the chimeric recPrP composed of the Syrian hamster residues 14 to 128 followed by sheep residues 141 to 234 of the R_154_ Q_171_ polymorphic haplotype as described before [7, 8]. The recPrP was prepared and its functionality was verified according to the method described by Wilham et al. [9].

### Study Design

The present study was performed in three stages in order to determine the reproducibility, stability and validity of RT-QuIC for use as a diagnostic test for human prion diseases:

1. To investigate the reproducibility of the RT-QuIC assay, we conducted two ring trial studies between different laboratories. Fifty-four CSF samples obtained from sCJD patients and 32 non-prion disease controls were examined in two laboratories (UMG, Thermo Fisher Scientific). In a second ring trial, one sCJD and five controls were investigated in four laboratories (UMG, Ilsong Institute of Life Science, Nagasaki University Graduate School of Biomedical Sciences, Western General Hospital, and University of Melbourne).
2. To calculate specificity, sensitivity and predictive values of the RT-QuIC assay, we analysed CSF samples from 110 prion disease patients, as well as from 400 control patients.
3. To determine the stability of RT-QuIC, sCJD CSF samples (*n* = 12) and control samples (*n* = 6) were incubated under short- and long-term storage conditions.
4. To assess the impact of blood contamination of CSF samples on the RT-QuIC response, we spiked CSF from sCJD (*n* = 6–8) and control samples (*n* = 6–8) with defined amounts of blood cells from control patients.
5. To investigate the impact of transportation on blood-contaminated samples, we spiked CSF from sCJD (*n* = 8) and control samples (*n* = 8) with a defined amount of blood cells from control patients and incubated the CSF samples for 0, 1, 3 and 8 days at room temperature.

### Statistical Analysis

In the first part of this study, inter-rater reliability within ring trials was calculated using the Fleiss’ kappa for multiple readers. A Fleiss’ kappa value of >0.60 was defined as substantial agreement, Fleiss’ kappa >0.80 as almost perfect agreement between raters.

To test the diagnostic validity, we established a training dataset consisting of 28 CJD patients and nine controls, which were not part of the present study. A Receiver operating characteristic (ROC) analysis was performed on this dataset, and the best cut-off value was estimated to be 10,000 rfu based on the Youden index. We thereby confirmed the analysis of McGuire et al. [4] in an independent dataset and applied the cut-off value of 10,000 rfu to this study.

For the analysis of the stability of RT-QuIC, a two-step approach was performed. First, positivity rates were calculated (defining a seeding efficiency of ≥10,000 rfu within 80 h as positive) and were compared between groups using ANOVAs. Agreement levels and Fleiss’ kappas were calculated in order to investigate the stability of positivity under different circumstances. Then, measurements of seeding efficiency were calculated for each individual sample defined by relative area under the curve (rel. AUC) and maximal ThT signal (maximum), and groups were compared using paired and unpaired *t* tests as appropriate. For graphical illustration, time series plots are presented at each step of the analysis, displaying means of seeding activity for each point in time (each measurement) in the respective groups. Thus, these illustrations show aggregated data for each point in time, but information about means of AUCs or individual maximums cannot be read out of these plots [7].

Sensitivity, specificity and predictive values of RT-QuIC were calculated for the entire dataset as well as for distinct subgroups. All analyses were conducted using Stata 12 software (StataCorp, College Town, US) and R 2.15.3 software.

### Analysis of CSF by ELISA for 14-3-3

Levels of 14-3-3 protein in CSF were determined by using the CircuLex 14-3-3 Gamma ELISA Kit (BIOZOL Diagnostica Vertrieb GmbH, Eching, Germany) according to a previously established protocol [7].

### Determination of Total Tau Level

CSF levels of total tau protein were measured using a commercially available ELISA kit (INNOTEST® hTAU Ag, Innogenetics). For the determination of tau levels, we followed the manufacturer’s instructions.

## Results

### Reliability of RT-QuIC Assay in Comparison to 14-3-3 and Tau Proteins

To investigate the reproducibility of PrP^Sc^ amplification via RT-QuIC assay, we initiated two ring trial studies and included comparison to other biomarker proteins (tau and 14-3-3). Results of inter-laboratory agreement were indicated as Fleiss’ kappa, which was calculated in a first ring trial with two partners as 0.75 (95 % CI: 0.40–1.00) revealing a substantial agreement, comparable to 14-3-3 and tau proteins (Supplement 1). A second ring trial with four raters showed an almost perfect agreement (Fleiss’ kappa = 0.83 (95 % CI: 0.40–1.00)), proving high reliability and reproducibility of the RT-QuIC assay (Supplement 1).

### Determination of the Specificity and Sensitivity of RT-QuIC Assay

**Fig. 1:**
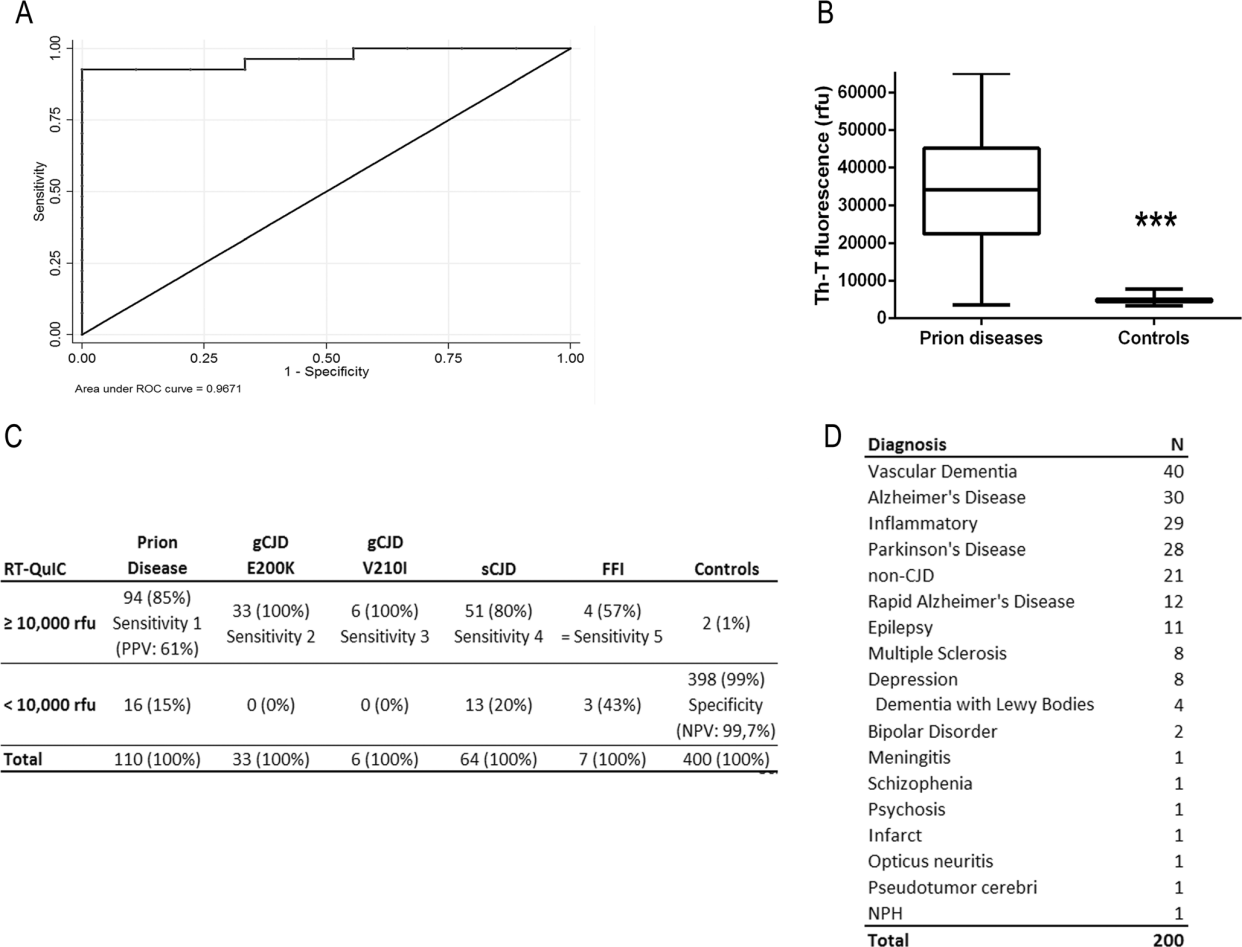
Analysis of the sensitivity and specificity of the RT-QuIC assay. RT-QuIC response was measured in rfu over a period of 80 h. **a** RT-QuIC signalling response was evaluated as a diagnostic test by using receiver operating characteristic (ROC) curves. The ROC curve of the training dataset is shown suggesting a cut-off at 10,000 rfu. **b** Prion diseases exhibited a median RT-QuIC signal of 35,000 rfu, while controls remained under 10,000 rfu. **c** Considering >50 % positive RT-QuIC replicates as positive for prion disease, we obtained a specificity of 99 % and an averaged sensitivity of 85 %. **d** Two hundred CSF samples, retrospectively tested by RT-QuIC, are summarized

Using a cut-off at 10,000 rfu/80 h (defined by Youden index[10]), an overall sensitivity of 85 % for all prion diseases and a specificity of 99 % could be achieved (Fig. 1a–c). Genetic CJD cases showed a sensitivity of 100 %, while analysis of sCJD and FFI CSF samples revealed a sensitivity of 80 and 57 %, respectively (Fig. 1c). The control cohort consisted of 200 patients with different diagnoses, e.g. AD, PD, IF etc. (Fig. 1d) and 200 prospectively tested patients.

However, we obtained two false positive results with a RT-QuIC response higher than 10,000 rfu in the control samples (Supplement 2). CSF sample 1 was from a patient originally clinically diagnosed as AD, while for sample 2, the diagnosis was still outstanding at the time of RT-QuIC analysis. Sample 1 showed no abnormalities with respect to 14-3-3 and total tau protein levels, while the second patient exhibited elevated tau and 14-3-3 levels, as well as a positive MRI, in keeping with prion disease. For patient 1, autopsy was not available and the patient has been lost to follow-up.

### Stability of the RT-QuIC Seeding Response with Long- and Short-Term CSF Storage Conditions

CSF samples derived from 12 sCJD and six control patients were stored in polypropylene tubes at room temperature or 4 °C for up to 8 days. To estimate the impact of long-term storage on the stability of RT-QuIC seeding response, we analysed sCJD (*n* = 12) and control (*n* = 6) samples, which were stored for 2–3, 5–6 or 8–9 years in a −80 °C freezer. Additionally, we subjected 12 sCJD and six control CSF samples to up to 16 repeated freezing and thawing cycles.

**Fig. 2:**
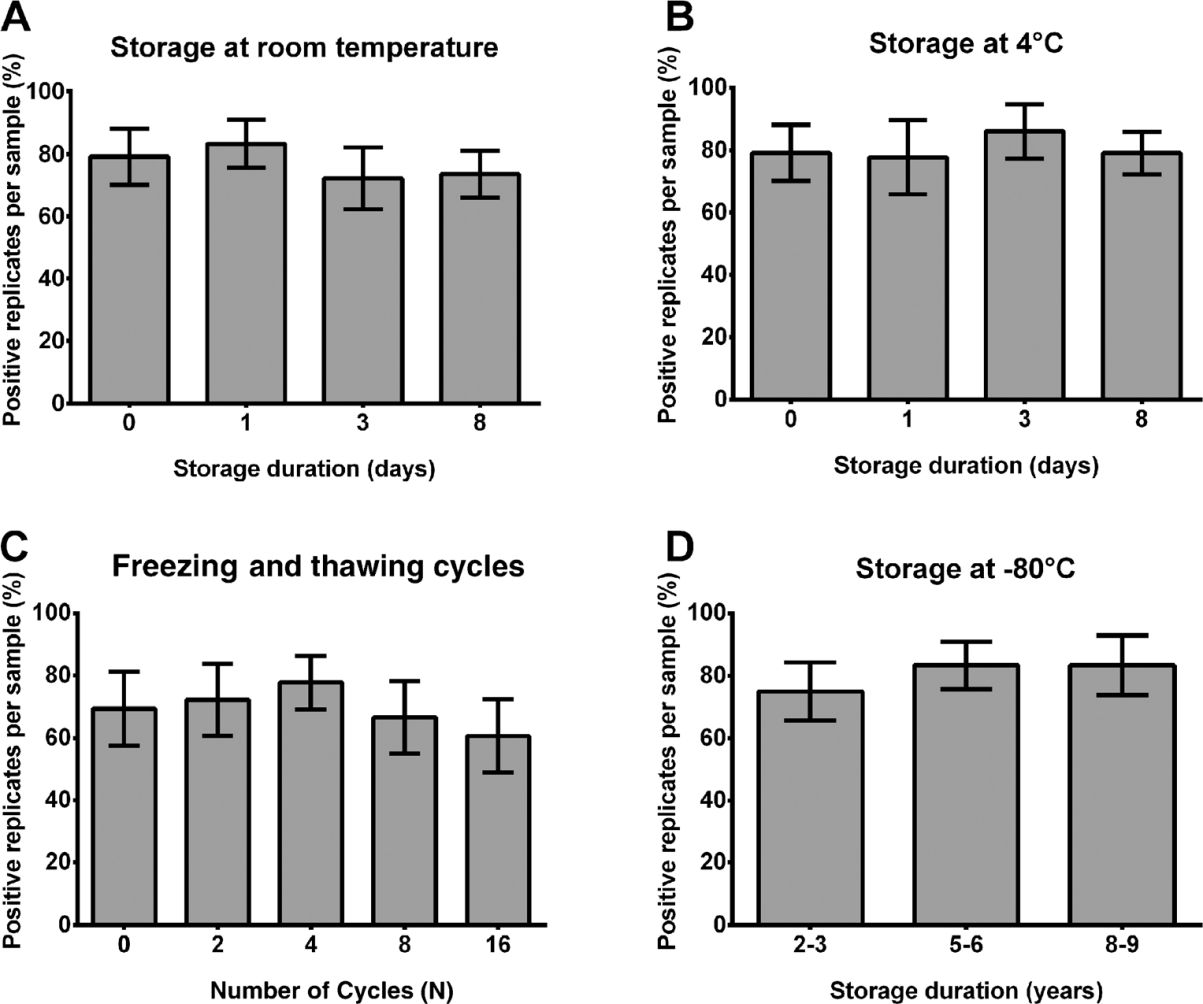
Effect of various storage conditions on the RT-QuIC signal response. RT-QuIC reactions seeded with CSF from sCJD patients were analysed under short-term storage conditions (8 days) at room temperature (RT) and 4 °C (**a**, **b**), after 16 repeated freezing and thawing cycles (**c**) and after long-term storage (up to 9 years) (**d**). There was no significant trend for decreasing positivity rates over time when **a** storing at room temperature (*p* = 0.337), **b** storing at 4 °C (*p* = 0.713) or **c** after 16 freezing and thawing cycles (*p* = 0.537) and **d** over several years of storage at −80 °C. Numbers of positive replicates were calculated for each sample in percent. Standard deviation of the mean from *n* = 12 patients per group was indicated by *error bars*. For comparison between groups, we used ANOVAs

We calculated for each indicated condition the percent of positive replicates per sample defined as a seeding response of more than 10,000 rfu (cut-off) in 80 h (3/3 = 100 %, 2/3 = 66 %, 1/3 = 33 % and 0/3 = 0 %), which is important for CJD diagnostic. Our data indicated that the PrP^Sc^ seed in the RT-QuIC reaction remained stable under the analysed short- and long-term storage conditions and was resistant to up to 16 repeated freezing and thawing cycles (Fig. 2a–d). Under short-term storage conditions, the temperature had no significant influence on the number of positive replicates.

**Fig. 3:**
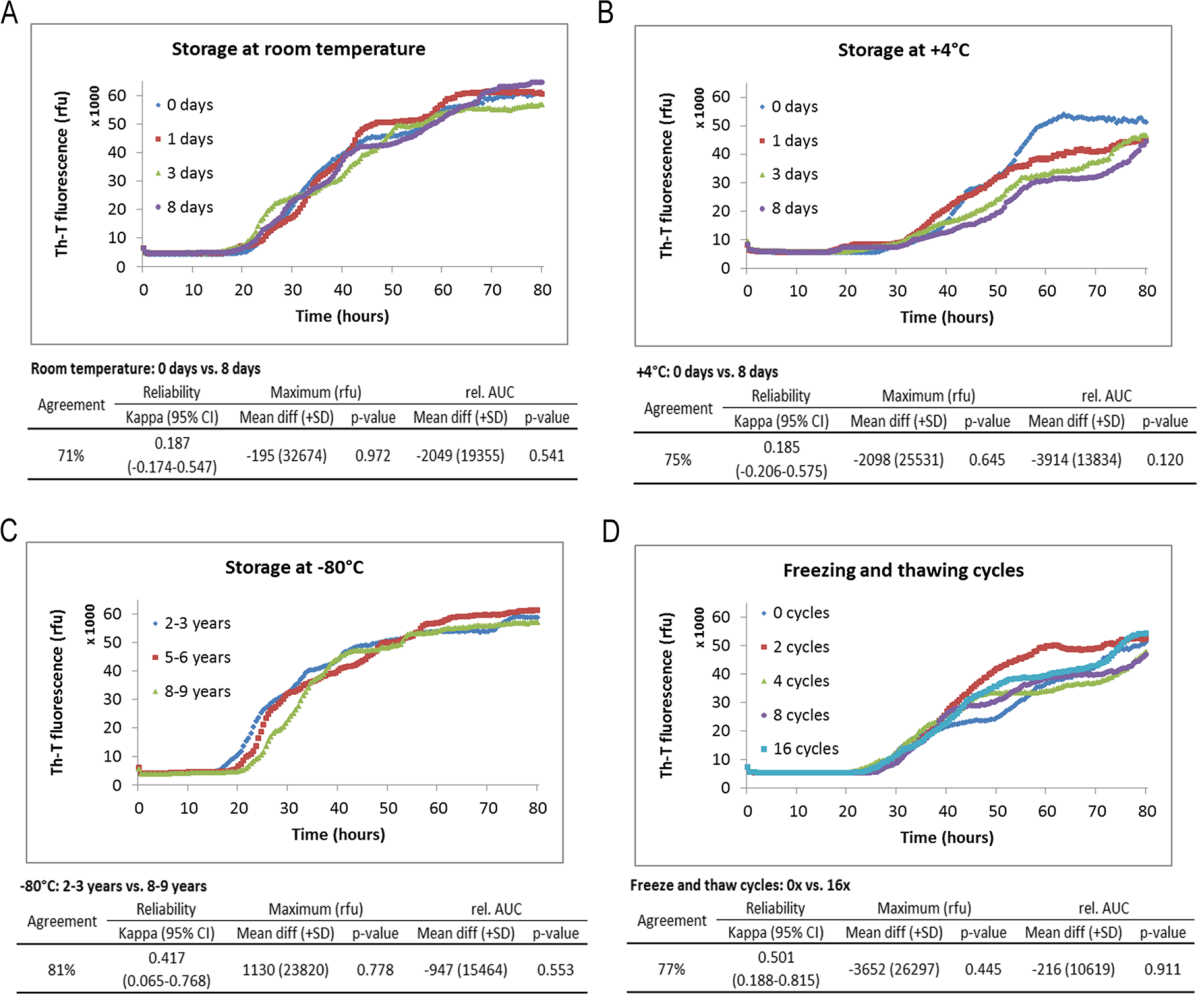
Time course of prion seeding activity in the CSF of sCJD patients after defined storage conditions. RT-QuIC reactions seeded with CSF from sCJD patients were analysed under short-term storage conditions (8 days) at room temperature (RT) and 4 °C (**a**, **b**) after long-term storage (up to 9 years) (**c**) and after 16 repeated freezing and thawing cycles (**d**). The PrP^Sc^ seeding efficiency was not significantly changed under these conditions. Graphically displayed are means of all positive replicates per group at each point in time. The absolute values for rel. AUC and signal maximum were shown for each group, and the *p* values were calculated for each comparative analysis. *p* values were obtained using paired or unpaired *t* tests for comparisons of maximum values and rel. AUCs

Subsequently, we analysed the RT-QuIC seeding efficiencies as a second parameter, which describes the intensity of the RT-QuIC signal, under the indicated storage conditions. We defined seeding efficiency as a combination of the following two parameters of interest: rel. AUC and the signal maximum of RT-QuIC response (maximum). Again, we observed that the seeding efficiency of PrP^Sc^ was not significantly influenced, neither under short-term nor under long-term storage conditions or after repeated freezing and thawing cycles of CSF samples (Fig. 3a–d).

Control samples without prion disease showed negative RT-QuIC responses (<10,000 rfu) under these conditions (data not shown).

### Influence of Blood Contamination on RT-QuIC Seeding Response in CSF

Since erythrocytes and platelets in human blood contamination of CSF may influence PrP^Sc^ seeding activity in the RT-QuIC assay, we spiked CSF derived from sCJD patients (*n* = 6–8) and non-CJD controls (*n* = 6–8) with different amounts of sonicated red blood cells from control donors (78, 313, 1250, 5000 or 10,000 erythrocytes/μL).

**Fig. 4:**
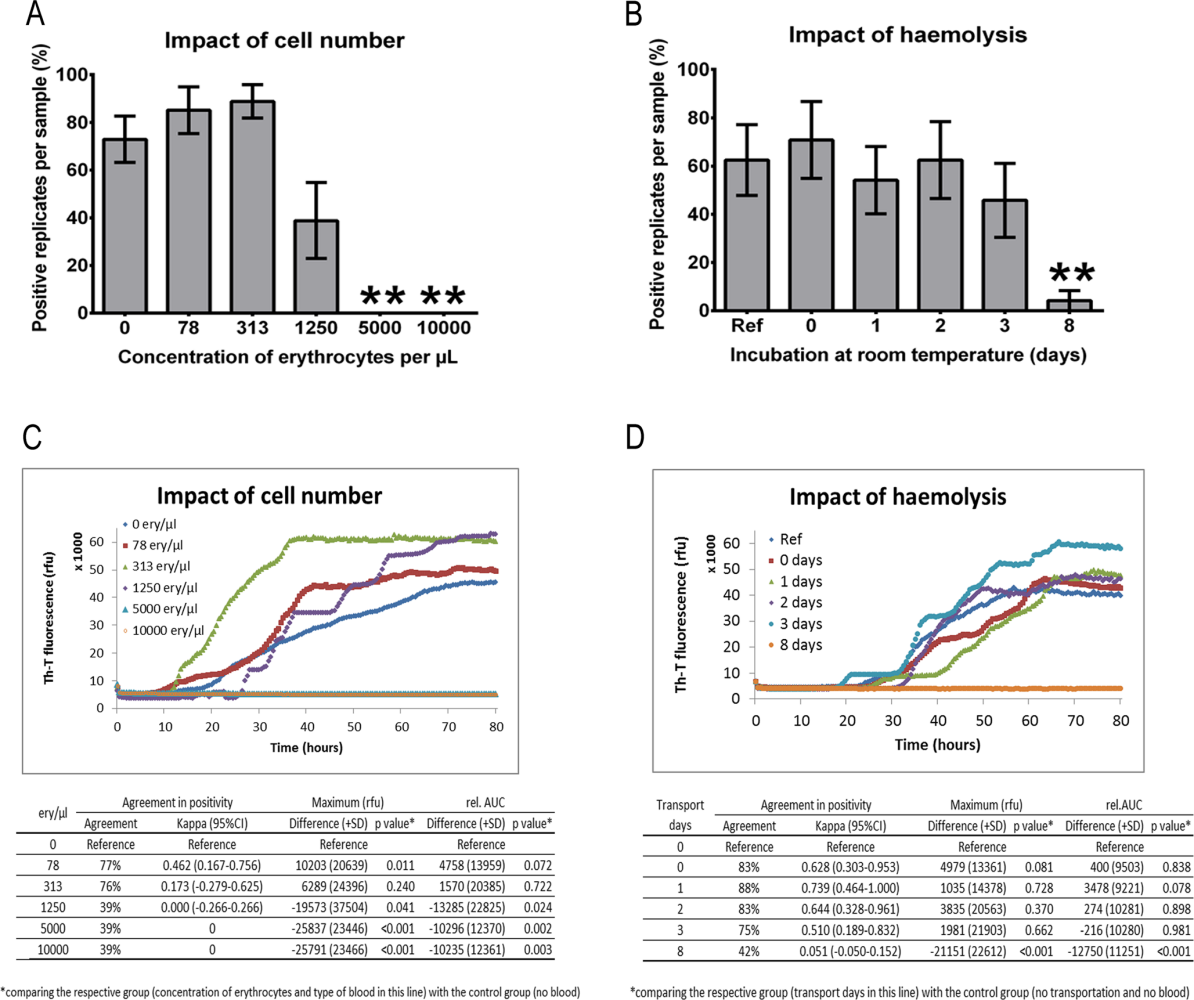
Influence of blood contamination on the RT-QuIC seeding response. CSF from sCJD patients (*n* = 6–8) was spiked with defined amounts of sonicated red blood cells. **a** The number of positive replicates was calculated and revealed that sCJD CSF samples spiked with more than 1250 cells/μL showed a false-negative RT-QuIC response. **b** CSF from sCJD (*n* = 8) patients was spiked with 5000 cells/μL and incubated at room temperature without sonication for 8 days showing that a significant inhibition via haemolysis of the RT-QuIC response started after 3 days. **c**, **d** Graphically displayed are means of all positive replicates per group at each point in time. Absolute values for rel. AUC and signal maximum were shown for each group, and the *p* values were calculated for each comparative analysis. All *p* values <0.05 were considered as significant

Our results indicated that an erythrocyte concentration higher than 1250 cells/μL showed a false-negative RT-QuIC response. Blood contaminations significantly influenced the number of positive replicates (Fig. 4a) as well as the signal intensity of the RT-QuIC response (Fig. 4c).

In contrast, erythrocyte concentrations below 1250 cells/μL are tolerable for diagnostics because they did not show any significant impact on the number of positive replicates and the intensity of the RT-QuIC signal (Fig. 4a, c). CSF from non-prion controls, spiked with blood always revealed a negative RT-QuIC seeding response (data not shown).

Subsequently, we simulated the impact of transportation on blood contaminated samples by spiking CSF samples from sCJD (*n* = 8) and control samples (*n* = 8) with a defined amount of blood cells (5000 cells/μL) from control patients. After 0, 1, 3 and 8 days, we centrifuged the samples to remove the blood cells. The haemolysis and release of inhibitory proteins, which influence the number of positive replicates and the intensity of the RT-QuIC signal significantly, started after 3 days resulting in a false-negative RT-QuIC seeding response in sCJD patients (Fig. 4b, d). For comparison, we used non-spiked CSF from sCJD patients as a reference.

## Discussion

The development of in vitro conversion assays, such as protein misfolding cyclic amplification (PMCA), enhanced quaking induced conversion (eQuIC) or RT-QuIC, exploit the self-propagating replication of the abnormally folded PrP^Sc^ to amplify miniscule amounts of PrP^Sc^ to a detectable level. Although these assays initially employed brain material, four recent studies demonstrated the capacity of RT-QuIC to replicate PrP^Sc^ from human CSF [4–7].

The use of RT-QuIC in routine CSF diagnostics for prion diseases requires standardization of sample treatments, definitions of cut-off values by analyzing a high number of samples and assessments of reproducibility and resistance of the RT-QuIC response to short- and long-term storage conditions; until now, only scarce information on these topics is available in the literature.

### Reproducibility of the RT-QuIC Assay Between Different Study Sites

To evaluate the inter-laboratory reproducibility of the RT-QuIC assay, we initiated ring trials between different laboratories. A first RT-QuIC ring trial between two different partners using a high number of samples (54 sCJD and 32 controls) indicated substantial agreement between raters (Fleiss’ kappa = 0.75 (95 % CI: 0.40–1.00)).

Even though the inter-laboratory agreement was relatively high, we intended to establish optimal conditions and standard operating procedures for the RT-QuIC method. The assay precision depends on various factors including (1) inter-laboratory variations in staff, protocols and substrates; (2) sensitivity of the assay to different CSF sample storage and shipping temperatures and (3) different fluorescent readers. By minimizing such variations, we achieved in a second ring trial an almost perfect agreement (Fleiss’ kappa = 0.83 (95 % CI: 0.40–1.00)) showing a high reproducibility of the RT-QuIC assay with improved standardization and supporting the potential of the method for routine diagnostic purposes.

### Sensitivity, Specificity and Predictive Values of the RT-QUIC Assay

We analysed CSF samples from a patient cohort consisting of 110 patients with prion disease (sCJD, gCJD and FFI) and 400 controls. When setting the cut-off at 10,000 rfu, we obtained a specificity of 99 % and a sensitivity of 85 % for all prion diseases. Among different prion diseases, the sensitivity varied from 100 % for gCJD, 80 % for sCJD and 57 % for FFI cases. Our data are in line with two previous studies, which reported a specificity of almost 100 % and a sensitivity between 80 and 90 % [4, 5]. For gCJD patients, Sano et al. reported sensitivities of RT-QUIC as follows: FFI (100 %), gCJD E200K (87 %) and gCJD V203I (100 %) [6]. Discrepancies for FFI patients may be explained by sampling errors because of the low number of specimens tested (7 versus 22 per group) and methodological differences [6, 7].

### Short- and Long-Term Stability of RT-QuIC Seeding Response in CSF

A general problem of biomarker-based diagnostics is the sensitivity of biomarker proteins to storage and shipment conditions. During shipment, CSF is subjected for a defined period of time (between 1 and 8 days) to different temperatures, which are between 20 and 25 °C. After arrival, CSF is usually stored at 4 °C before analysis. Samples, which are used for research, may undergo numerous cycles of freezing and thawing or will be stored for years at −80 °C.

For simulation of these conditions, we explored the effect of short-term storage (up to 8 days at room temperature or at 4 °C), long-term storage (up to 8–9 years at −80 °C), as well as the influence of 16 repeated freezing and thawing cycles on the RT-QuIC seeding response. Our data demonstrated that the PrP^Sc^ seed in CSF of sCJD patients resisted the variations posed with across the analysed conditions, and we did not obtain any loss of sensitivity or specificity or in relation to the intensity of the RT-QuIC responses.

Compared to other biomarker proteins such as tau or Aβ42, which become degraded in particular under higher temperatures during storage [11–13], the proven stability of RT-QuIC assay is a clear advantage with respect to diagnostic purposes.

### Influence of Blood Contamination on the RT-QuIC Response in CSF of sCJD Patients

Contamination with blood cells, such as erythrocytes, may influence the RT-QuIC response. In routine diagnostic laboratory practice, between 5 and 10 % of the CSF samples are contaminated with blood cells during the lumbar puncture. Therefore, there is a need to define clear guidelines for pre-analytical sample handling. Our data revealed an artificial inhibition of the PrP^Sc^ seeding activity which resulted in a false-negative RT-QuIC response in prion disease patients. Our findings may explain the variable sensitivity of the RT-QuIC assay (between 80 and 90 %) among different laboratories [4, 5]. The obtained data indicated that a blood cell contamination higher than 1250 cells/μL may inhibit the RT-QuIC reaction, when all cells were completely haemolysed by sonication. A contamination with <1250 cells/μL can be considered as unproblematic. In CSF samples (not older than 3 days after the lumbar puncture), a centrifugation step before freezing is recommended to remove blood cells. After 3 days, haemolysis of blood cells was increased to such an amount that no reliable results could be obtained. Therefore, it is strongly suggested to exclude these samples from further diagnostic analysis.

In conclusion, our data emphasize that RT-QuIC analysis of CSF is a robust and very useful tool for prion diagnosis and should become part of the clinical workup in rapidly progressive dementia cases.

## Electronic supplementary material

Below is the link to the electronic supplementary material.

Supplement 1 **Reproducibility of RT-QuIC in comparison to 14-3-3 and tau proteins.** CSF from sCJD patients and control donors were analysed in two ring trials. Results of inter-laboratory agreement were shown as Fleiss’ kappa. In a first ring trial with two partners RT-QuIC assay revealed a substantial agreement, which is comparable to 14-3-3 and tau. A second ring trial with more standardized condition showed an almost perfect agreement (Fleiss’ kappa =0.83). (GIF 43 kb)

High resolution image (TIFF 3756 kb)

Supplement 2 **Time course of prion seeding activity of two control samples showing a potential false positive RT-QuIC response.** Patient 1 showed no abnormalities, the second patient exhibited an elevated tau and 14-3-3 level as well as a CJD typical MRI which are indices for a prion disease. (GIF 403 kb)

High resolution image (TIFF 2144 kb)

## Abbreviations

CI: Confidence interval
CNS: Central nervous system
CSF: Cerebrospinal fluid
diff: Difference
FFI: Fatal familial insomnia
PrP^C^: Cellular prion protein
PrP^Sc^: Scrapie prion protein
PPV: Positive predictive value
NPV: Negative predictive value
ref: Reference
rcf: Relative centrifugal force
rel. AUC: Relative area under the curve
ROC: Receiver operating characteristic
rpm: Rounds per minute
RT-QuIC: Real-time quaking-induced conversion
rfu: Relative fluorescence units
CJD: Creutzfeldt-Jakob disease
gCJD: Genetic CJD
sCJD: Sporadic CJD
SD: Standard deviation
SEM: Standard error of the mean
