# Supplementary figures and images for: Stability and Reproducibility Underscore Utility of RT-QuIC for Diagnosis of Creutzfeldt-Jakob Disease
Source: Mol Neurobiol. 2015 Apr 1;53(3):1896–904. doi: 10.1007/s12035-015-9133-2 (PMC4789202; doi:10.1007/s12035-015-9133-2)

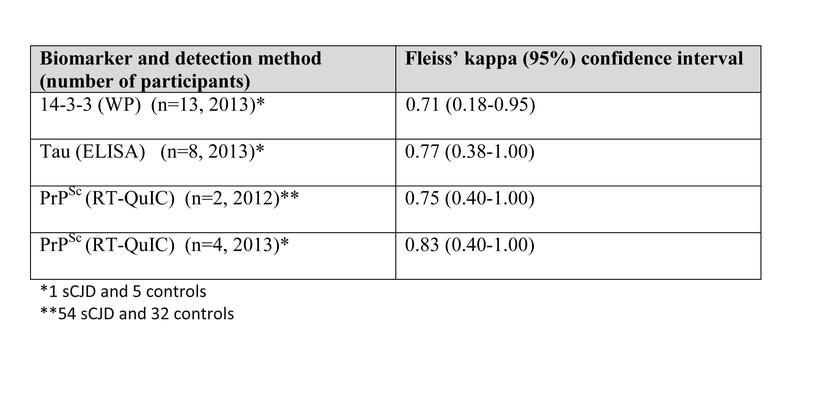

Supplement: Supplementary file 1 — Reproducibility of RT-QuIC in comparison to 14-3-3 and tau proteins. CSF from sCJD patients and control donors were analysed in two ring trials. Results of inter-laboratory agreement were shown as Fleiss’ kappa. In a first ring trial with two partners RT-QuIC assay revealed a substantial agreement, which is comparable to 14-3-3 and tau. A second ring trial with more standardized condition showed an almost perfect agreement (Fleiss’ kappa =0.83). (GIF 43 kb) [file 12035_2015_9133_Fig5_ESM.gif]

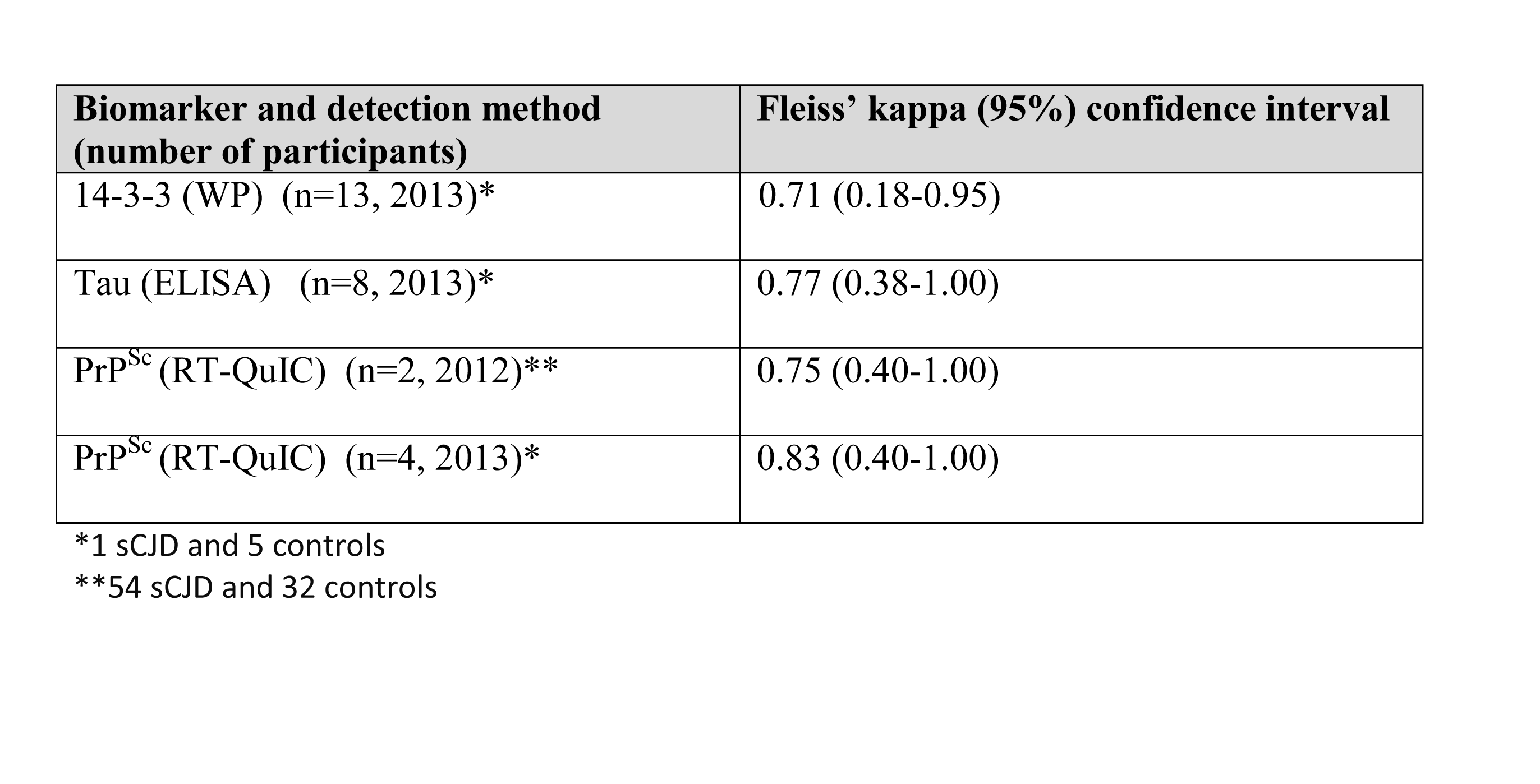

Supplement: Supplementary file 2 — High resolution image (TIFF 3756 kb) [file 12035_2015_9133_MOESM1_ESM.tif]

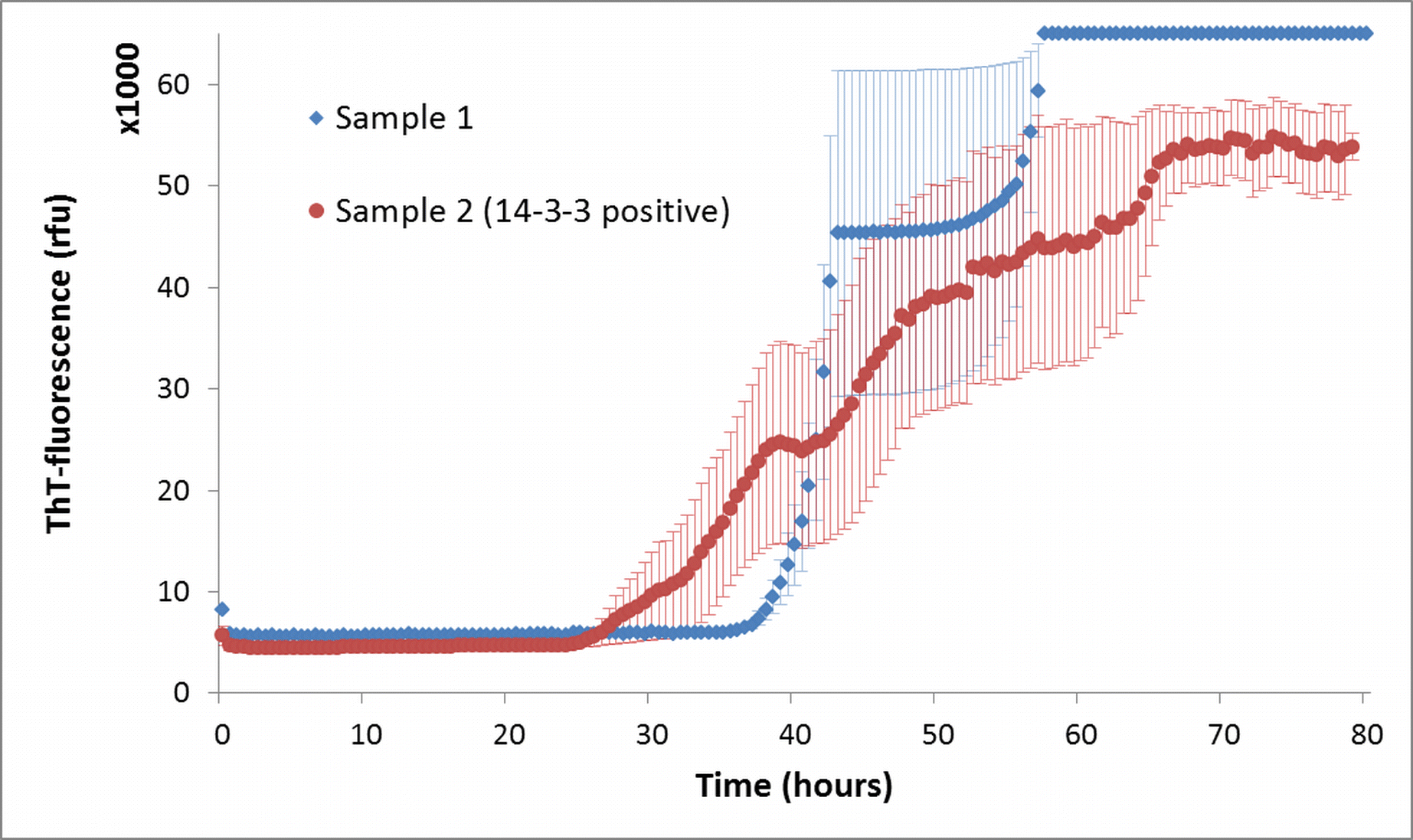

Supplement: Supplementary file 3 — Time course of prion seeding activity of two control samples showing a potential false positive RT-QuIC response. Patient 1 showed no abnormalities, the second patient exhibited an elevated tau and 14-3-3 level as well as a CJD typical MRI which are indices for a prion disease. (GIF 403 kb) [file 12035_2015_9133_Fig6_ESM.gif]

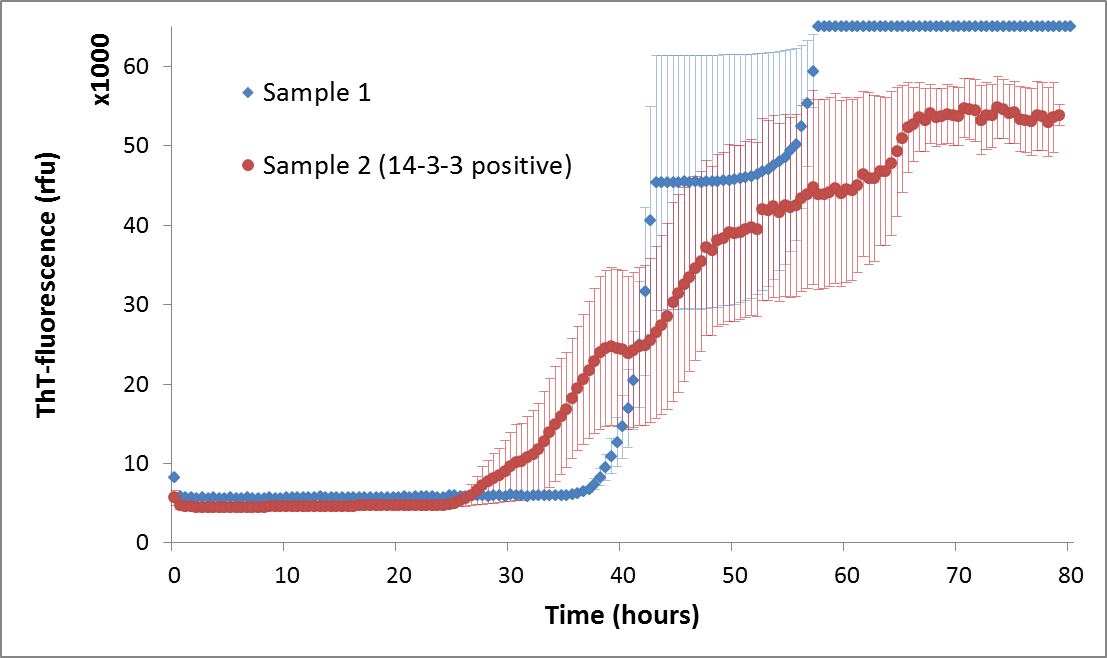

Supplement: Supplementary file 4 — High resolution image (TIFF 2144 kb) [file 12035_2015_9133_MOESM2_ESM.tif]
